# Effects of high-quality nursing care for patients with lung cancer during the perioperative period: A protocol of systematic review of randomized controlled trials

**DOI:** 10.1097/MD.0000000000018132

**Published:** 2019-11-27

**Authors:** Xia Yu, Jun Liu

**Affiliations:** a Department of Acupuncture and Rehabilitation; b Department of Neonatology, The Second Affiliated Hospital of Shaanxi University of Chinese Medicine, Xianyang, China.

**Keywords:** effectiveness, high-quality nursing care, lung cancer, randomized controlled trial

## Abstract

**Background:**

This study will explore the effects of high-quality nursing care (HQNC) for patients with lung cancer (LC) during the perioperative period (PPP).

**Methods:**

A literature search will be performed at Cochrane Library, MEDLINE, EMBASE, Chinese Biomedical Literature Database, and China National Knowledge Infrastructure since its inception until October 1, 2019. All electronic databases will be searched with no restrictions of language and publication status. Two authors will perform study selection, data collection, and study quality assessment, respectively. We will use RevMan 5.3 software for statistical analysis.

**Results:**

This study will summarize the latest evidence on assessing the depression, anxiety, quality of life, and adverse events of HQNC in patients with LC during PPP.

**Conclusion:**

The results of this study may provide helpful evidence of HQNC on psychological effects in patients with LC during PPP.

**PROSPERO registration number:**

PROSPERO CRD42019155982.

## 1 Introduction

Lung cancer (LC) is one of the most causes of cancer-related death around the world.^[1–3]^ It has been estimated that about 85% LC is non-small-cell LC.^[4–5]^ The 5-year overall survival for such patients is less than 15%, and even no more than 5% patients reach to IV stage of LC.^[6]^ The most common therapies for LC, including chemotherapy, radiotherapy, and surgery are reported to effectively treat LC, especially for surgery.^[7–16]^ However, most patients who received surgery also experience more psychological disorders, such as depression and anxiety.^[17–20]^ Advanced nursing care (high-quality nursing care [HQNC]) has been reported to effectively prevent such condition in patients with LC post-surgery.^[18,21–24]^ Unfortunately, no study has systematically assessed the psychological effects of HQNC for patients with LC during perioperative period (PPP). Therefore, this study will first evaluate the psychological effects of HQNC for patients with LC during PPP.

## 2 Methods

### 2.1 Eligibility criteria

#### 2.1.1 Types of studies

All relevant randomized controlled trials (RCTs) on investigating the psychological effects of HQNC in patients with LC during PPP will be included. However, we will exclude any other studies, such as nonclinical studies, and noncontrolled trails.

#### 2.1.2 Types of participants

We will include any patients with LC during PPP who are clinically diagnosed as psychological disorder with no restrictions of regions, race, and gender.

#### 2.1.3 Types of interventions

In the experimental group, all patients must receive HQNC for the psychological disorders.

In the control group, all patients can undergo any interventions, except any forms of HQNC.

#### 2.1.4 Types of outcomes

The primary outcome is depression, which is measured by the Hamilton Depression Rating Scale or any relevant scales.

The secondary outcomes comprise of anxiety, as measured by the Hamilton Anxiety Rating Scale or other tools; health-related quality of life, as assessed by any associated scales or scores; and any expected or unexpected adverse events.

### 2.2 Search methods for identification of studies

We will search the following electronic databases of Cochrane Library, MEDLINE, EMBASE, Chinese Biomedical Literature Database, and China National Knowledge Infrastructure from its inception to October 1, 2019. No restrictions of language and publication status will be applied to any electronic databases. Additionally, we will also search unpublished and ongoing trials on the clinical trial registry, conference proceedings, and dissertations. The sample of search strategy of the Cochrane Library is presented in Table 1. We will also adapt similar search strategies to other databases.

**Table 1:**
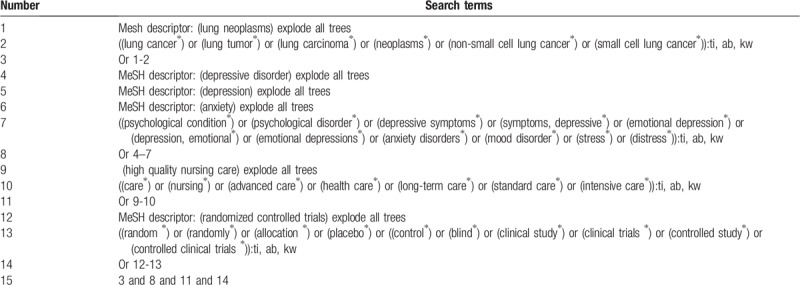
Detailed search strategy for Cochrane Library.

### 2.3 Data collection

#### 2.3.1 Study selection

Two authors will independently screen all citations and references using predefined standard eligibility criteria. The whole study selection consists of 2 stages: first checking titles and abstracts, and second reading the full texts. Any divergences in reviewing will be solved by consensus with the help of a third author if needed. The study selection process is presented in the flowchart.

#### 2.3.2 Data extraction

Two authors will perform data extraction independently in accordance with pre-designed data extraction sheets. The extracted data will comprises of study title, first author, baseline characteristic data, diagnostic criteria, exclusion and inclusion criteria, study methods, intervention details, outcomes, and information on the methodological quality for each trial. A third author will solve disagreements in data extraction between 2 authors.

#### 2.3.3 Missing data dealing with

If we encounter missing or unclear data, we will try to contact corresponding authors for additional information. We will analyze available data if we can not obtain that additional information. In addition, we will also report potential impact of those missing or unclear data.

### 2.4 Assessment of risk of bias for included studies

Two authors will assess the risk of bias for all included studies using the Cochrane Risk of Bias Tool, respectively. For each included study, we will provide a description for all aspects assessed, and each one will be categorized as low, unclear, and high risk of bias. A third author will be available to solve any divergences and to make a final judgment if necessary.

### 2.5 Data synthesis

In this study, RevMan 5.3 software will be used for data synthesis and data analysis. All continuous values will be calculated with mean difference or standardized mean difference and 95% confidence intervals (CIs). All dichotomous data will be expressed as risk ratio and 95% CIs. We will use *I*^2^ statistic to identify heterogeneity among eligible studies. The values of *I*^2^ statistic are interpreted as follows: *I*^2^ ≤50% means acceptable heterogeneity and a fixed-effect model will be used, while *I*^2^ >50% indicates obvious heterogeneity, and a random-effect model will be utilized. When sufficient eligible studies are available on the same outcome measurements with acceptable heterogeneity, meta-analysis will be planed to perform. We will carry out subgroup analysis if obvious heterogeneity is identified. If there is still obvious heterogeneity after subgroup analysis, we will report outcome results as narrative summary based on the Guidance on the Conduct of Narrative Synthesis in Systematic Reviews.^[25]^

### 2.6 Subgroup analysis

We will carry out subgroup analysis in according to the different managements, study quality, and outcome assessments.

### 2.7 Sensitivity analysis

A sensitivity analysis will be performed to check the robustness of outcome results by excluding studies with high risk of bias.

### 2.8 Reporting bias

We will look for potential reporting bias using the funnel plot^[26]^ and Egger regression^[27]^ if at least 10 included studies are included.

### 2.9 Ethics and dissemination

This study will not analyze individual patient data, thus, no ethic approval is needed. This study will be published at a peer-reviewed journal.

## 3 Discussion

This study is intended to summarize the evidencing investigating the effectiveness and safety of HQNC on psychological effects in patients with LC during PPP. The results of this study will provide rigorous summary evidence and will inform our understanding of HQNC on psychological effects in patients with LC during PPP across published RCTs. This study may provide helpful evidence for both clinical practice and future research. However, there are still limitations associated to the different heterogeneity of the eligible studies.

## Author contributions.

**Conceptualization:** Xia Yu, Jun Liu.

**Data curation:** Xia Yu, Jun Liu.

**Formal analysis:** Xia Yu, Jun Liu.

**Investigation:** Jun Liu.

**Methodology:** Xia Yu, Jun Liu.

**Project administration:** Jun Liu.

**Resources:** Xia Yu, Jun Liu.

**Software:** Xia Yu, Jun Liu.

**Supervision:** Jun Liu.

**Validation:** Xia Yu, Jun Liu.

**Visualization:** Xia Yu, Jun Liu.

**Writing – original draft:** Xia Yu, Jun Liu.

**Writing – review & editing:** Xia Yu, Jun Liu.
